# Spontaneous coronary artery dissection: dissecting an underdiagnosed problem

**DOI:** 10.1007/s12471-025-01992-x

**Published:** 2025-10-28

**Authors:** Deborah N. Kalkman, Arja S. Vink, Marcel A. M. Beijk, Bert-Jan H. van den Born, Jurriën M. ten Berg, Fatih Arslan, Yolande Appelman, Eric Wierda

**Affiliations:** 1 Department of Clinical and Experimental Cardiology, Heart Center, Amsterdam Cardiovascular Sciences, Amsterdam UMC—University of Amsterdam, Amsterdam, The Netherlands; 2 Department of Internal Medicine, Division of Vascular Medicine, Amsterdam UMC—University of Amsterdam, Amsterdam, The Netherlands; 3 Department of Cardiology, St. Antonius Hospital, Nieuwegein, The Netherlands; 4 Cardiovascular Research Institute Maastricht, Maastricht, The Netherlands; 5 Department of Cardiology, Leiden University Medical Center, Leiden, The Netherlands; 6 Department of Cardiology, Dijklander Ziekenhuis—Hoorn & Purmerend, Hoorn, The Netherlands

**Keywords:** Acute coronary syndrome, Spontaneous coronary artery dissection, Fibromuscular dysplasia, Hypertension, Peripartum, Antiplatelet therapy

## Abstract

Spontaneous coronary artery dissection (SCAD) occurs in 1–4% of acute coronary syndromes (ACS). In SCAD, an intramural hematoma compresses the true lumen of the coronary artery, leading to ischemia and, even acute myocardial infarction.

Approximately, 90% percent of SCAD patients are premenopausal women without classical risk factors for atherosclerosis. The gold standard for diagnosis is invasive coronary angiography and optical coherence tomography or intravascular ultrasound can be useful tools to confirm the diagnosis. Coronary intervention with stent placement is generally not recommended unless there is complete occlusion of the coronary artery with ongoing ischemia. In the acute phase, antiplatelet therapy and beta-blockers are advised, which are usually continued for life. Despite medical treatment, 10–20% of SCAD patients experience a recurrence within 4 years. Nearly two-thirds of SCAD patients have fibromuscular dysplasia (FMD) based on CT angiography. Current treatment recommendations are based on expert opinion. Therapy and follow-up are advised to include at least one antiplatelet agent, a beta-blocker, screening for FMD, cardiac rehabilitation and among patients with left ventricular systolic dysfunction ACE inhibitor or aldosterone receptor blocker. Randomized controlled trials have been initiated to evaluate the treatment effects of beta-blocker and antiplatelet therapy in SCAD patients.

## Introduction

**Fig. 1:**
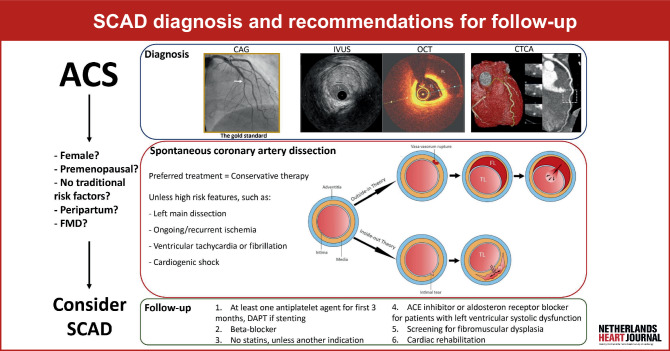
*SCAD* spontaneous coronary artery dissection, *CAG* coronary angiogram, *IVUS* intravascular ultrasound, *OCT* optical coherence tomography, *CTCA* computed tomography coronary angiography, *FL* false lumen, *TL* true lumen. Adaptation of figures by Adlam et al. [24], Dang et al. [12]

Approximately 1–4% of all acute coronary syndromes (ACS) are caused by a spontaneous coronary artery dissection (SCAD) (Fig. 1;[1]). SCAD results in myocardial ischemia due to lumen narrowing or occlusion caused by an intramural hematoma, in contrast to plaque rupture or erosion in atherosclerotic coronary artery disease (CAD). SCAD predominantly affects women (90% of patients) who are relatively young and lack traditional atherosclerotic risk factors [2]. In fact, among women under 50 years of age, SCAD may account for nearly a quarter of all ACS cases [2].

Despite increasing scientific interest in SCAD, recognizing this disease remains challenging [3–6]. Here, we present an updated review summarizing current literature on SCAD (‘What is known and what is new?’) and anticipated future data (‘What is next?’).

## What is known and what is new?

### Mechanism and causes

SCAD is caused by a spontaneous loss of vessel wall integrity [7]. Two hypotheses have been proposed for the pathological process the ‘inside-out’ and the ‘outside-in’ hypotheses. The ‘inside-out’ hypothesis suggests that due to a tear of the intima, blood enters the subintimal space from the true lumen, forming an intramural hematoma (IMH). The ‘outside-in’ hypothesis suggests a de novo IMH in the media due to a hemorrhage of the vasa vasorum [1]. Irrespective of the mechanism of IMH formation, the final common pathway for ACS is obstruction of the coronary blood flow by the expanding hematoma or dissection flap.

It is suggested that increased shear stress of the coronary vessel wall, together with increased catecholamines, and in some cases elevated intra-abdominal pressure, leads to SCAD, especially if there is a pre-existing arteriopathy such as fibromuscular dysplasia (FMD) [8]. Up to two-thirds of SCAD patients have FMD, [7, 9] a non-inflammatory, non-atherosclerotic syndrome, which leads to arterial stenosis, occlusions, aneurysms, dissections, and tortuosity [10]. The most affected arteries are the renal and carotid arteries [10]. There is no clear cause, but FMD is associated with a single-nucleotide variant of the PHACTR1-gene [9]. which has been associated with migraine headaches, FMD, and cervico-cerebral artery dissections [11]. Other causes of SCAD are much less frequent but can include connective tissue disorders, systemic inflammatory diseases, pregnancy-related SCAD, or hormone-associated SCAD [12].

In 3.5% of SCAD patients, there is a suggestive pathological genetic variant, mostly found in the genes associated with other syndromes such as Ehlers-Danlos, Loeys-Dietz, or polycystic kidney disease [13, 14]. A recent analysis of healthy controls, individuals with sporadic SCAD, and thirteen families with SCAD-affected patients revealed that common genetic variants (7 single-nucleotide variants) may predispose to SCAD [15].

SCAD is associated with the peripartum period and progesterone and estrogen therapy [16]. These hormones may cause changes in the vascular function and structure. Progesterone is hypothesized to increase the deposition of non-collagen proteins and estrogen, decreasing elastin and collagen deposition, resulting in weakening of the vessel wall [17, 18]. Pregnancy-related SCAD occurs between early pregnancy and 18 months postpartum, with the highest incidence within 6 weeks after delivery. Pregnancy-related SCAD constitutes 5–17% of all SCAD cases [19]. Increased cardiac output and circulating volume, and acute hemodynamic changes all increase the chance of SCAD [20].

Of the traditional risk factors for atherosclerosis, only hypertension is associated with the occurrence of SCAD [19]. Approximately 30% of SCAD patients have hypertension, which—in part—may be related to the concurrent presence of renovascular FMD [21].

### Classification of SCAD

**Fig. 2:**
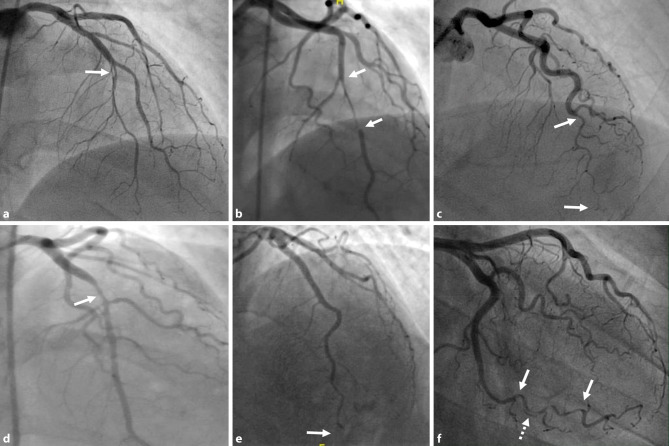
Four different subtypes of SCAD by Adlam et al. [24]. Panel A: Type 1 SCAD, panel B: Type 2A SCAD, panel C: Type 2B SCAD, panel D: Type 3 SCAD, panel E: Type 4 SCAD, and panel F: Intermediate Type 1/2 SCAD

The current classification [22–24] in four groups is as follows (Fig. 2):

1. Multiple radiolucent lumina.
2. Diffuse stenosis of varying severity and length, which may be bordered by normal artery segments proximal and distal to the IMH (type 2A) or may extend to the apical tip of the artery (type 2B).
3. Focal or tubular stenosis (typically < 20 mm) that mimics atherosclerosis and requires intracoronary imaging to confirm the diagnosis.
4. Total distal occlusion of the coronary vessel or other occlusions not meeting the criteria for types 1 to 3.

**Fig. 3:**
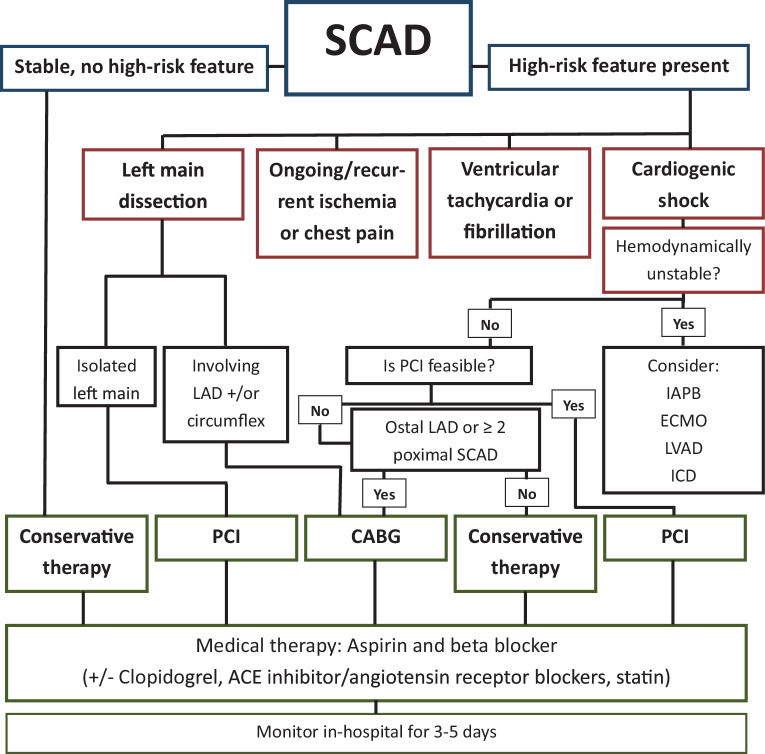
SCAD management algorithm. Adapted from Saw et al. [1] *SCAD* spontaneous coronary artery dissection, *LAD* left anterior descending artery, *PCI* percutaneous coronary intervention, *IABP* intra aortic balloon pump, *ECMO* extracorporeal membrane oxygenation, *LVAD* left ventricular assist device, *ICD*: implantable cardioverter defibrillator, *CABG* coronary artery bypass grafting

Type 2 is the most frequently observed type of SCAD (± 60%).

### Prevalence

The exact prevalence of SCAD is unknown, due to underreporting of this cause of ACS [1, 25]. Most reports acknowledge SCAD as the cause of ACS in up to 4% of ACS cases, and in 0.5% as a cause of sudden cardiac death. In women under 50 years of age, SCAD is reported to be the cause of ACS in 22–43% of cases [21]. SCAD is rarely observed in patients younger than 25 or older than 80 years.

### Signs and symptoms

The clinical presentation of SCAD patients is similar to that of ACS, with symptoms of chest pain with radiation, nausea, and vomiting [21, 26]. In 25–50% of cases, ST-segment elevation can be found on the electrocardiogram. Up to 90% of SCAD patients present with ACS, approximately half of patients present with ST-segment elevation MI (STEMI), and about half with non-ST-elevation ACS [27]. Emotions, physical stressors, but also drugs such as amphetamines and cocaine, childbirth and Valsalva maneuver could trigger SCAD. Nine percent of patients present with ventricular tachycardia/fibrillation [21]. Incidence of cardiogenic shock has been reported between 1.2% and 15.9% of the cases, with a higher percentage in peripartum SCAD (20–24%). These peripartum patients are reported to be younger and more likely to have connective tissue disorders than patients without cardiogenic shock.

### Diagnostics and acute treatment

Laboratory testing can show elevated troponin levels. Coronary angiography remains the diagnostic tool of first choice [24]. Recent publications have reported computed tomography coronary angiography (CCTA) as a non-invasive alternative technique for invasive angiography that might be preferable over angiography in stable patients without STEMI or rise of serum troponin level or worsening chest pain because of less chance of complications related to the coronary angiography [28, 29]. However, sufficient spatial and temporal resolution is needed for adequate assessment of SCAD on CCTA [30].

It remains challenging to recognize SCAD, as it can mimic atherosclerotic disease or vasospastic angina [22]. Coronary artery spasm may resemble type 2 SCAD on invasive angiography. Administration of intracoronary nitroglycerin can help discriminate between the two entities, as coronary artery spasm usually resolves with nitroglycerin. Type 3 SCAD can look like focal atherosclerosis. In these cases, optical coherence tomography (OCT) or intravascular ultrasound (IVUS) can allow for better insight into the cause of the ACS [2]. Though OCT use should be performed by experienced operators, since contrast injections can worsen vessel occlusion. The left anterior descending artery, particularly in the mid or distal section of the coronary artery, is affected in about 50%; the circumflex coronary artery in about 30%; and the right coronary artery in about 25%. Multivessel location occurs in about 15%, and the left main coronary artery is affected in about 4% [21]. SCAD of the left main and left anterior descending artery (LAD) has been observed more frequently in shock patients [31]. Distal vessels are more commonly affected than the proximal vessels. In a propensity-matched analysis of 11 patients with SCAD and 11 healthy patients with coronary artery assessment with CCTA more tortuosity and a sharper angle between the LAD and the adjacent arterial branch was observed in the SCAD group [32].

In 70–97% of cases, a conservative approach results in complete healing of the vessel wall [33]. Only in patients with ongoing ischemia or cardiogenic shock should revascularization be considered (Fig. 3, [34]). Percutaneous coronary intervention (PCI) is associated with more complications and less angiographic success as compared with PCI for atherosclerotic lesions. Unintended wiring of the false lumen may occur, and balloon dilatation and/or stent placement in the false lumen can lead to propagation of hematoma and vessel occlusion requiring emergent coronary artery bypass grafting (CABG). Moreover, after healing of the IMH stent, malposition can lead to increased risk of stent thrombosis. CABG is technically challenging in dissected vessels; it is a temporizing measure and long-term graft patency is a concern [16]. A scientific statement from the American Heart Association states that prolonged hospitalization of 3 to 5 days for monitoring of the hemodynamics and ischemia (risk of dissection extension or new recurrent SCAD) in patients is justified as part of the conservative treatment strategy [2]. The 2023 European Society of Cardiology (ESC) guidelines for the management of acute coronary syndromes do not give recommendations for the duration of monitoring in-hospital for SCAD patients [35].

### Clinical outcomes

In-hospital recurrent myocardial infarction occurs in 1.9% of conservatively treated patients and 17.9% of patients undergoing PCI during hospitalization [36]. Post-discharge major adverse cardiac event (MACE, consisting of death, recurrent acute myocardial infarction, unplanned revascularization, stroke/transient ischemic attack, and congestive heart failure) rates were reported in approximately 3% within 30 days in the non-PCI group and 26.4% in the PCI-treated group [36]. The recurrence rate for SCAD is 10–20% within 3 years [21, 37, 38]. In 75–90% of the recurrent cases, the dissection is observed in another coronary segment. Hypertension is associated with a higher recurrence of SCAD [39]. Moreover, patients with pregnancy-related SCAD have worse clinical outcomes than patients with non-pregnancy-related SCAD. One in seven patients with pregnancy-related SCAD experiences a new SCAD during a subsequent pregnancy [20, 40].

Long-term outcomes have been described in the largest SCAD registry from Canada, including 750 patients, which showed a mortality rate of 0.8%, recurrent myocardial infarction in 9.9% and major adverse cardiac events in 14.0% during 3‑year follow-up [36]. Congestive heart failure was reported in 1.9% of patients and severe ventricular arrhythmia in 4.4% of patients at 3‑year follow-up [36].

### Follow-up and long-term treatment

The 2023 ESC guidelines for the management of acute coronary syndromes recommend, in the absence of randomized controlled trials in SCAD patients, they receive the same pharmacological therapy as other ACS patients [35]. Moreover, SCAD patients with left ventricular dysfunction should be treated according to standard heart failure guidelines [41], and those receiving stents should be treated according to guidelines for PCI management. Based on observed longitudinal associations in registry data, the use of aspirin and beta-blockers is recommended.

The DISCO (DIssezioni Spontanee COronariche) registry showed that dual-antiplatelet therapy was associated with higher adverse cardiac events compared to single antiplatelet therapy, surprisingly driven by higher numbers of non-fatal MI and unplanned PCI. It is important to note that the DISCO trial was a relatively small retrospective cohort with differences between the groups (e.g., more type I SCAD) [42]. Lifelong beta-blocker therapy, if tolerated, can be justified based on a retrospective study in which its use was associated with lower rates of SCAD recurrence [43]. Cardiac rehabilitation should be considered in all patients, and special emphasis should be placed on assessing mental health in patients after SCAD [44–46]. Large regional and global variations in treatment of SCAD patients have been observed, with regard to medication prescriptions and FMD screening [41]. Currently, no guideline recommendations are available to patients with peripartum SCAD and following pregnancies. Though a viewpoint written by Tweet et al. stated that women of childbearing age after SCAD should be counseled to avoid future pregnancy [47]. The authors based this advice on the high recurrence rates in SCAD patients, lack of prediction models to predict who is at risk of recurrence, and due to the fact that the clinical course of pregnancy and postpartum SCAD is more severe than other SCAD [47]. As this advice has a great impact on the lives of SCAD patients, we suggest that shared decision making should be the aim of pregnancy counseling.

Patients should be screened at the outpatient clinic for the presence of FMD. In the Netherlands this is preferably done by an internal medicine doctor in a specialized FMD center. Screening is mostly performed with CT angiography to detect stenosis of the arteries, such as the carotid and renal arteries. Genetic testing for FMD is not performed routinely because of the lack of clinical implications. Follow-up of FMD consists of strict blood pressure management [10].

## What is next?

### International registries

The European Society of Cardiology has launched a registry (EURObservational Research Programme (EORP) SCAD) in approximately 30 countries and 120 sites to prospectively collect data on 500 SCAD patients and gather a retrospective dataset of 500 SCAD patients to better understand the epidemiology, clinical presentation, management, and outcomes of SCAD patients. The International Spontaneous Coronary Artery Dissection (iSCAD) Registry (clinical trials identifier NCT04496687) aims to enroll 1000 SCAD patients in 26 sites, mostly from the United States of America to evaluate best practices.

The Mayo Clinic has a clinical trials program specifically for SCAD, including a “Virtual” Multicenter SCAD Registry (NCT01429727), and an investigation of the role of the sympathetic nervous system in SCAD (NCT05699200), differences between pregnancy-related SCAD and non-pregnancy-related SCAD (NCT03390998) and genetic investigations (NCT01427179).

### Randomized controlled trials

Unfortunately, the Statin and Angiotensin-converting Enzyme Inhibitor on Symptoms in Patients With SCAD (SAFER-SCAD, NCT02008786) trial was terminated due to slow recruitment of patients. This illustrates the difficulty of conducting RCTs in SCAD patients but emphasizes the need for collaborative initiatives.

The Spanish BA-SCAD trial (Randomized Study of Beta-Blockers and Antiplatelets in Patients With Spontaneous Coronary Artery Dissection, ClinicalTrials.gov ID NCT04850417) will evaluate the treatment effect of beta-blocker combined with either short-term (1 month) or long-term duration (12 months) dual antiplatelet therapy in 600 SCAD patients in a 2 × 2 design (patients will be randomized 1:1/1:1) [48].

The international, prospective APT-SCAD trial will evaluate the effect of a moderate intensity antiplatelet treatment (APT) strategy (3 months aspirin followed by cessation of APT) in comparison to an intensive treatment regimen (3 months aspirin and clopidogrel followed by 9 months clopidogrel) in conservatively managed SCAD patients with respect to MACE at 12 months, defined as cardiovascular mortality, myocardial infarction, recurrent SCAD, unplanned coronary revascularization and ischemic stroke/transient ischemic attack.

### Dutch initiatives

The Vrouwenhart (SCAD) challenge from University Medical Center (UMC) Utrecht, is a yearly contest for medical students to initiate SCAD research. Hart voor Vrouwen (Heart for Women, www.hartvoorvrouwen.nl), a research organization from RadboudUMC also increases knowledge and awareness of SCAD. The patient collective Vrouwenhart (https://vrouwenhart.nl) continues to pursue gender/sex-related differences awareness among physicians, researchers, patients, and the general public. The recently launched website https://www.scadnederland.nl is a patient initiative to provide evidence-based SCAD information to patients in collaboration with healthcare professionals.

The Dutch Fibromuscular Network, consisting of vascular medicine specialists from Maastricht, Utrecht, Amsterdam, Rotterdam, Nijmegen, Sittard, Tilburg and the Hague have, in close collaboration with the radiologists in their hospitals, drawn up a general protocol for the evaluation of patients who are suspected of having FMD. This uniformity in patient approach will thus allow the establishment of a Dutch FMD Registry. The Network is actively supported by patients with FMD (www.fmdgroep.nl).

### Knowledge gaps and future research

Predictors of SCAD and the use of CCTA as a diagnostic approach would be topics of interest for future research. Moreover, optimal medical treatment and follow-up management should be studied to evaluate the safety and efficacy of current strategies. Gaining more insight in which patients are at risk of recurrence and understanding when full recovery of the dissected coronary arteries has been achieved would be of importance to better guide treatment after SCAD.

As only limited randomized data are currently available in SCAD patients, much more research and collaboration is needed.

## Conclusion

In conclusion, SCAD accounts for a significant proportion of young women who present with ACS. SCAD is classified into four subtypes; in all, compression of the true coronary vessel lumen is caused by an intramural hematoma. CCTA can play a role in the diagnosing SCAD, although invasive coronary angiography remains the gold standard. Intravascular imaging may be useful in cases of diagnostic uncertainty. There are genes that are associated with SCAD, and as nearly half of SCAD patients seem to be affected with FMD, screening for FMD should follow in the outpatient clinic. Current medical therapy consists of antiplatelet therapy and beta-blockers. Hypertension management should be a main focus in the follow-up of these patients.

Future collaborative research initiatives will allow for better understanding of diagnosis and treatment options for SCAD and community initiatives will continue to increase awareness of SCAD in the general public.
